# Circulation of small ruminant lentivirus in endangered goat and sheep breeds of Southern Italy

**DOI:** 10.1016/j.heliyon.2024.e33906

**Published:** 2024-06-28

**Authors:** Angela Ostuni, Sara Albarella, Luca Tassoni, Mariagiulia Pugliano, Emanuele D'Anza, Maria Antonietta Crudele, Francesca Ciotola, Maria Serena Beato, Valentina Iovane, Stefano Cecchini Gualandi, Raffaele Frontoso, Jolanda De Vendel, Vincenzo Peretti, Alfonso Bavoso

**Affiliations:** a Department of Sciences, University of Basilicata, Via dell’ Ateneo Lucano 10, 85100, Potenza, Italy; b Department of Veterinary Medicine and Animal Production, University of Naples Federico II, Via Delpino 1, 80137, Napoli, Italy; c National Reference Laboratory for Ruminant retroviruses, Istituto Zooprofilattico Sperimentale dell'Umbria e delle Marche (IZSUM), Via G. Salvemini 1, 06126, Perugia, PG, Italy; d Dipartimento di Agraria, Università degli Studi di Napoli Federico II, Via Università 100, 80055, Portici, NA, Italy; e Istituto Zooprofilattico Sperimentale del Mezzogiorno, Via Salute, 2, 80055, Portici, NA, Italy; f OneHEco APS, 84047, Capaccio Paestum, SA, Italy

**Keywords:** Small ruminant lentivirus, Endangered breeds, ELISA test, Genotypes, Biodiversity

## Abstract

According to the Domestic Animal Diversity Information System (DAD-IS) of the FAO, Italy has one of the largest numbers of local small ruminant breeds among European countries. In Southern Italy, namely the Campania Region, Bagnolese and Laticauda sheep breeds and Cilentana goat breeds are considered endangered according to the DAD-IS. Conservation of endangered animal breeds is a goal of the European Union (EU). However, the role of infectious diseases as risk factors for endangered breeds has rarely been considered. Small ruminant lentiviruses (SRLV) infect sheep and goats, causing slow-progressive, persistent, and debilitating diseases that can lead to animal death and productivity loss. In this study, we investigated the presence of SRLV in Bagnolese, Laticauda, and Cilentana breeds using a commercial ELISA in parallel with an in-house ELISA. The results of the two tests were in good agreement (Cohen Kappa 0.84, 95 % CI = 0.76–0.93). Discrepancies between the two tests were resolved using western blotting. In total, 430 samples were tested (248 Bagnolese, 125 Laticauda, and 57 Cilentana). The apparent prevalence rates were 12.5 %, 6.4 %, and 1.7 % in Bagnolese, Laticauda, and Cilentana, respectively. In the molecular analysis of 11 proviral partial sequences, subtypes B2 and A24 were identified in two Bagnolese herds. Owing to the beneficial role of sheep and goat breeding in marginal areas, it is important to screen the entire population and implement control/eradication of SRLV infections in conjunction with each conservation program.

## 1 Introduction

Italy is an extremely heterogeneous territory with different habitats populated by numerous native animal breeds of different species. In Southern Italy, namely the Campania Region, 13 autochthonous breeds belonging to five different species coexist [1]. Small ruminant species include the Bagnolese and Laticauda sheep breeds and the Cilentana goat breeds. All breeds were registered in the official herdbook managed by Associazione Nazionale della Pastorizia (AssoNaPa; National Shepherding Association). Sheep and goat breeding in marginal areas contributes to land management, local economy enhancement, and biodiversity conservation.

Bagnolese sheep, called *Malvizza,* are derived from crosses between indigenous sheep and the Barbaresca breed imported into the Campania region from North Africa [1]. It is primarily distributed in the Picentini Mountains, Alburni, Vallo di Diano, and Piana del Sele areas and marginally in the hills of Caserta and Benevento. According to official data (AssoNaPa, 2023), in 2022 there were 9584 animals (390 males and 9184 females) were enrolled in the birth register and distributed across 119 farms. Today, there has been a shift to a semi-wild and semi-extensive system with feed supplementation, with preference given to the use of grains produced in areas close to the rearing areas that affect the organoleptic characteristics of the milk produced as little as possible. The average milk production per lactation is 105 kg (unpublished data), which is mainly used for dairy transformation into Pecorino Bagnolese cheese.

Laticauda is a dual purpose (milk and meat) sheep breed, and its morphological characteristic is a 'broad tail.' This probably originated from crosses between the local Apennine and Barbaresca sheep. It is bred in the Campania region, particularly in the Benevento, Caserta, and Avellino provinces. The herd book contains 1487 animals (79 males and 1408 females) enrolled and distributed across 58 farms (official data by AssoNaPa, 2023). It is reared in medium hills and herds of between 20 and 200 heads. In the past, the principal management technique was transhumance. Currently, herds are generally sedentary and use the territory resources for grazing. Laticauda milk is mainly used for Laticauda Sannita Pecorino cheese production, whereas lamb meat is appreciated for its low cholesterol content and the absence of a wild flavor.

The Cilentana goat is an ancient breed, tracing back to the 4th century B.C. Currently, three different breeds are recognized, namely Fulva, Nera, and Grigia, which probably originated from the cross-breeding of local populations with the Derivata di Siria, Garganica, and Maltese. The particular conformation of the environment in which this breed developed (the Rocky Mountains and steep coasts) made it a strong goat that is suitable for wild or semi-wild breeding to be fed on pasture. All Cilentana goat breeds are dual-purpose goats (meat and milk), and milk is used mainly for cheese production (“*cacioricotta*,” a typical local cheese). Cilentana goats are raised in the Salerno Province, within the territory of the National Parks of Cilento, Vallo di Diano, and Alburni. Currently, 1940 subjects (1476 females and 60 males) are enrolled in the book, distributed across 58 farms (official data by AssoNaPa, 2023).

The control of infectious diseases and the development of effective diagnostic tests are important tools for the preservation of native breeds and species that share the same living areas.

Small ruminant lentiviruses (SRLV) infect sheep and goats and cause slow-progressive, persistent, and debilitating diseases [2]. Under certain circumstances, failure of many organs can occur, which can lead to animal death. The infection results in productivity losses in both ovine and caprine herds [3]. The international animal trade has been adversely affected. The SRLV group comprises two genetically, structurally, and antigenically related viruses, Visna/Maedi Virus (MVV) and Caprine Arthritis Encephalitis Virus (CAEV), which are members of the genus Lentivirus of the family *Retroviridae*. Previously, MVV was considered specific to sheep, whereas CAEV was believed to infect goats specifically. However, recent studies have demonstrated that these two viruses are part of the genetic continuum of lentiviral species [2,4] and that cross-species transmission is possible [[5], [6], [7], [8]].

SRLVs are classified into five genotypes or clades with subtypes [9,10]. The original MMV corresponds to genotype A (subtypes A1–A24), whereas genotype B (subtypes B1–B5) corresponds to the original CAEV [2]. In addition to these two genotypes, which are widely distributed and comprise most subtypes, geographically restricted genotypes C, D, and E have also been reported [6,[11], [12], [13]]. A limited number of SRLV investigations have been performed in Italy, particularly in the southern region [[14], [15], [16]]. Since the virus permanently infects the host and no vaccine exists, the only way to control the infection diffusion is to identify positive animals and isolate or cull them. Several control and eradication programs have been established worldwide, including those in Italy, with varying degrees of success [[17], [18], [19], [20]].

Only a small proportion of animals infected with SRLV develop clinical signs; therefore, diagnosis is primarily based on serology, which is the most reliable and cost-effective diagnostic method for identifying persistently SRLV-infected animals (OIE, 2021, https://www.oie.int/fileadmin/Home/eng/Health_standards/tahm/2.07.02-03_CAE_MV.pdf/). Several commercial indirect or competitive ELISA have been developed, and three different recombinant protein antigens have been selected and used in most cases: the capsid protein p25 (CA), transmembrane protein gp46 (TM), and gp135 envelope protein. In this respect, we developed an in-house indirect ELISA with access to reliable and cost-effective tests for routine applications [21].

The aim of the present study was to serologically screen Bagnolese and Laticauda sheep herds and Cilentana goat herds from the Campania region (Southern Italy) to map the circulation of SRLV and identify the circulating subtypes. To the best of our knowledge, this report is one of the few SRLV screenings regarding autochthonous endangered sheep and goat breeds.

## 2 Materials and methods

### 2.1 Farms and animals

Seventeen farms, all registered at the breeding association (AACM) and located in the provinces of Avellino, Benevento Salerno, and Caserta (Campania, Southern Italy), were selected for this study using geo-localization data matched with the rearing areas of the three breeds.

Blood samples for genetic and immunological analyses were obtained from 430 animals, including 125 Laticauda sheep (120 females and 5 males), 248 Bagnolese sheep (239 females and 9 males), and 57 Cilentana goats (55 females and 2 males). All sampled animals were registered in the official herdbook (AssoNaPa 2023). All procedures were approved by the Ethical Animal Care and Use Committee of the University of Naples Federico II (Prot. Nr. PG/2022/0146435 del 05/12/2022).

### 2.2 ELISA

Serum samples were serologically analyzed in parallel using a commercial indirect assay coated with a panel of synthetic peptides from SRLV structural proteins comprising surface glycoprotein (gp135, SU), transmembrane glycoprotein (gp46, TM), and capsid protein (p25/p28, CA) (ID Screen MVV-CAEV Indirect Screening test; IDvet Innovative Diagnostics, Grabels, France) and an in-house indirect assay, as previously reported [21]. In a comparative analysis of seven commercially available serological tests on Belgian sheep and goats, the IDvet screening ELISA showed a sensitivity of 100 % for both species and specificities of 97.8 % and 97.6 % for sheep and goats, respectively [22]. The in-house ELISA showed a 96.0 % sensitivity and a 96.0 % specificity in a preliminary analysis [21].

The commercial assay was performed according to the manufacturer's instructions. An in-house test was performed, as previously described. Maxi Binding Immuno plates (SPL Life Sciences Co., Korea) were coated with polypeptides obtained by recombinant DNA technology consisting of a genotype A P25 protein (P25_1) (molecular mass 26194,6 Da), a genotype B P25 fragment (P25_2frag) (molecular mass 19000 Da) and a double strain polypeptide derived from the transmembrane protein (TM_1_2) (molecular mass 29840.7 Da) containing sequences related to both genotype A and B. Polypeptides were used at a fixed mass ratio of 1:1:2 for P25_1, P25_2frag and TM_1_2. Protein G Peroxidase (Merck KGaA, Darmstadt, Germany) was added as a secondary antibody, and tetramethylbenzidine (Merck KGaA, Darmstadt, Germany) was used as a substrate. In both cases, the optical density was measured at a wavelength of 450 nm using a microplate reader (Multiskan GO Microplate Spectrophotometer, Thermo Fisher Scientific).

### 2.3 Western blot analysis

Western blot analysis was performed as previously reported [23] with some modifications. A mixture of P25_1, P25_2frag, and TM_1_2 in a mass ratio of 1.5 μg: 0.5 μg: 0.01 μg was used, respectively. The polypeptides were resolved onto 4–15 % sodium dodecyl sulfate–polyacrylamide gels electrophoresis and electrotransferred to nitrocellulose membranes (Amersham Bioscience, Buckinghamshire, UK). The membranes were blocked in saturation buffer (2 % non-fat dried milk in PBS with 0.05 % Tween 20, PBST) for 1 h at 20 °C and then probed for 2 h at 37 °C with sera 1:200 in PBS-T. Membranes were washed with PBS-T and then incubated at room temperature for 1 h with Protein G-peroxidase 1:6000 and signal visualized by ECL™ Western Blotting Detection Reagents (GE Healthcare, Chicago, IL, USA), using a Chemidoc™ XRS detection system equipped with Image Lab Software for image acquisition (BioRad, Hercules, CA, USA). The signals were visually classified into four levels (not visible, very weak, weak, and strong). A sample was considered positive if at least one band was either weak or strong.

### 2.4 DNA analysis

DNA was extracted from the blood of 76 animals using a Wizard DNA extraction kit (Promega, Madison, WI, USA) following the manufacturer's instructions and quantified using an Eppendorf BioPhotometer (Eppendorf AG, Hamburg, Germany). DNA showed the following requirements: yield >30 ng/μL, 260/280 ratio >1.7, and 260/230 ratio >1.8.

The genetic study was based on an 800 bp long fragment corresponding to the partial *gag-pol* gene from proviral SRLV DNA, as previously described [5]. The nested-PCR protocol previously described by Grego et al. (2007) [13] was performed using GoTaq® G2 DNA Polymerase (Promega, Madison, WI, USA). All the PCR products were visualized on 1.8 % agarose gels in Tris-Acetate-EDTA (TAE) buffer. The 800 bp fragment was extracted from the agarose gel using a commercial kit (Canvax, Cordoba, Spain) and then sequenced by the Sanger method using an external service (BMR Genomics Srl, Padova, Italy). From all 800 bp-long PCR fragments (n = 25), 11 sequences were obtained and deposited in GenBank (accession numbers: OR420124 - OR420134).

### 2.5 Phylogenetic analysis

To determine the genotypes and subtypes of the acquired sequences, a dataset consisting of representative samples of Italian circulating genotypes that were previously identified and described in the literature was used [16,24,25]. Additionally, we included a few sequences that were found to be the most similar to those under study using BLAST, particularly blastn and tblastn, to retrieve the most similar sequences using a nucleotide or its amino acid translation as a query, respectively [26]. All sequences were aligned using MAFFT v7 [27], and the alignment was manually curated based on the amino acid sequence in MEGA X [28].

After preliminary phylogenetic analysis, the final dataset was enriched with all retrievable sequences belonging to the same subtype as our samples to obtain a final dataset consisting of 320 sequences of approximately 800 bp, corresponding to the *gag-pol* encoding region.

Phylogenetic analysis was performed using the Maximum Likelihood method implemented in IQtree v.2.1.3, with GTR + G + I substitution model and 1000 ultrafast bootstrap (UFBoot) replicates [29,30]. We also performed an analysis using the Maximum Likelihood method in PhyML 3.1 with 100 nonparametric bootstrap replicates to confirm the topology relative to the studied sequences [31].

The phylogenetic trees were visualized using Figtree v1.4.4 (http://tree.bio.ed.ac.uk/); the final image was edited using Inkscape.

Genetic distances (p-distances) were calculated using MEGA X and were used to calculate the similarity percentage between sequences.

## 3 Results

### 3.1 Serological analysis- ELISA/Western blot

The overall agreement between the in-house test and the IDvet screening ELISA was 97 % (417 results in agreement in 430 tested samples) with a Cohen kappa = 0.84 (95 % CI = 0.76–0.93) [32].

**Fig. 1:**
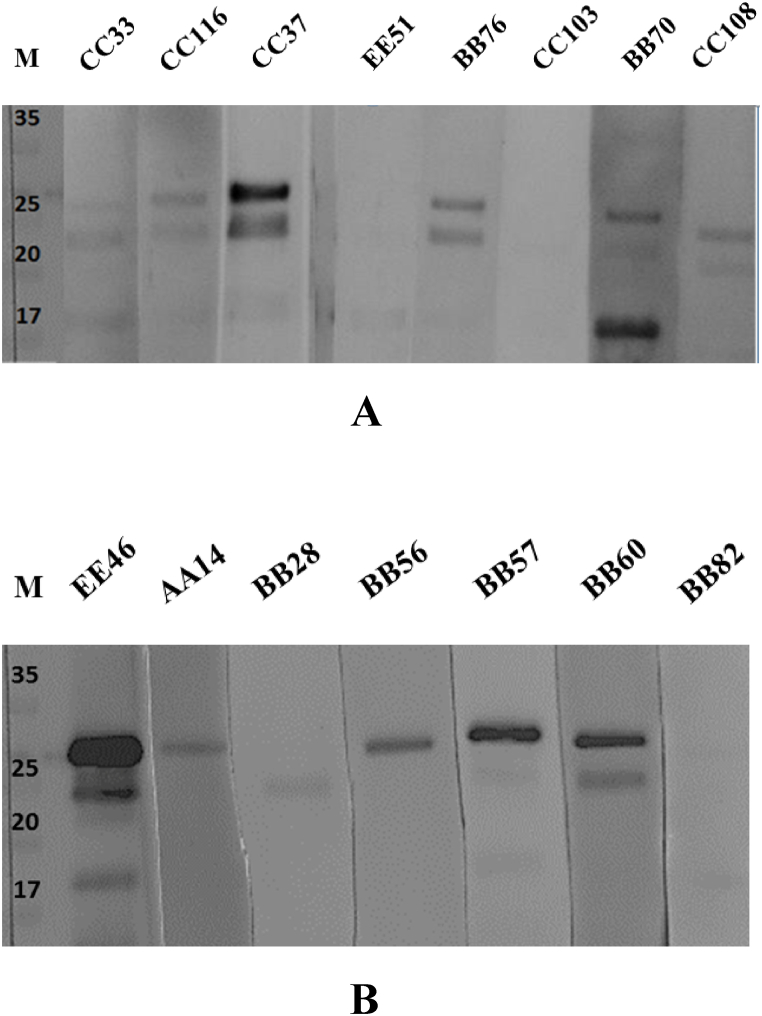
Western blot analysis of sera from Bagnolese and Laticauda sheeps and Cilentana goats. (A) CC33 (Bagnolese), CC116 (Laticauda), CC37 (positive control, Bagnolese), EE51 (Cilentana), BB76 (Bagnolese), CC33 (Laticauda), BB70 (Bagnolese) CC108 (Laticauda); (B) EE46 (positive control, Cilentana goat), AA14 (Bagnolese), BB28 (Bagnolese) BB56 (Bagnolese), BB57 (Bagnolese), BB60 (Bagnolese), BB82 (Bagnolese). M indicates protein molecular weight markers. The original blots are shown in Supplementary Fig. S1.

The 13 samples for which the two tests yielded different results were subsequently analyzed using western blotting (Fig. 1A and B). Seven out of 13 samples were classified as positive according to the western blotting results, following previously reported criteria (Section 2.3).

Therefore, we concluded that 31 were positive in the 248 Bagnolese sheep tested with an apparent prevalence of 12.5 %; in this breed, 50.0 % of the herds had positive animals. In the 125 Laticauda sheep, eight positives were found, giving an apparent prevalence of 6.4 %; in this case, of the five tested herds, one showed positive animals. A single positive was found in the two tested herds among the 57 Cilentana goats (1.7 % apparent prevalence). The overall data for each herd are presented in Table 1.

**Table 1:** Overall results of the ELISA screening and molecular analysis for each farm.

Province	Farm	Animals enrolled in the Birth Register^a^	Animals tested	ELISA Positives	Sequenced Samples	Breed
**Avellino**	1	130	20	6 (30.0 %)		Bagnolese
	2	52	19	0		
	3	46	38	1 (2.6 %)		
	4	121	29	0		Laticauda
**Salerno**	1	121	22	0		Bagnolese
	2	111	22	0		
	3	150	40	14 (35.0 %)	6	
	4	55	28	0		Cilentana
	5	30	29	1 (3.4 %)		
**Benevento**	1	52	20	0		Bagnolese
	2	113	22	8 (36.4 %)	5	
	3	41	21	0		
	4	13	24	2 (8.3 %)		
	5	25	15	0		Laticauda
	6	29	22	8 (36.4 %)		
	7	2	28	0		
**Caserta**	1	184	31	0		Laticauda

a The number of animals enrolled in the Birth Register may be lower than the number of tested animals because certain animals were in the phase of inscription to the register.

### 3.2 Molecular analysis

**Fig. 2:**
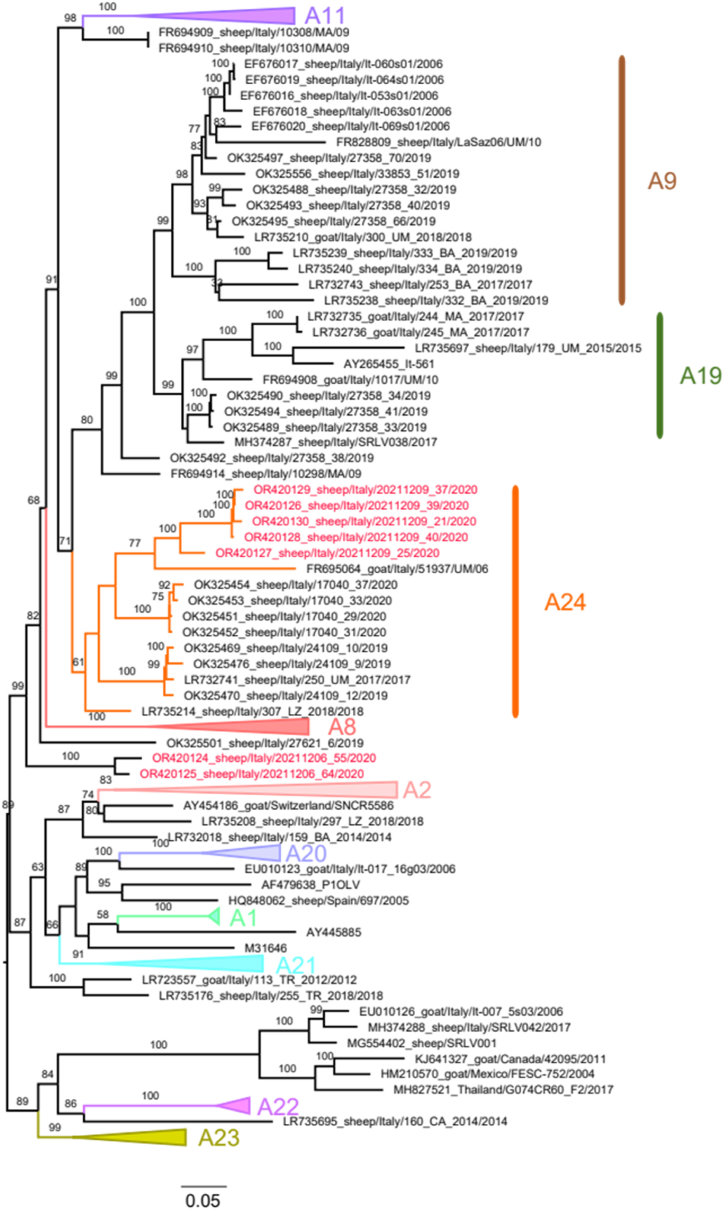
Maximum likelihood phylogenetic tree of genotype A samples. Samples of this study are colored in red and the corresponding subtype is highlighted in orange (A24). To enhance visualization, other subtypes were collapsed. Only bootstrap values above 60 are displayed.

**Fig. 3:**
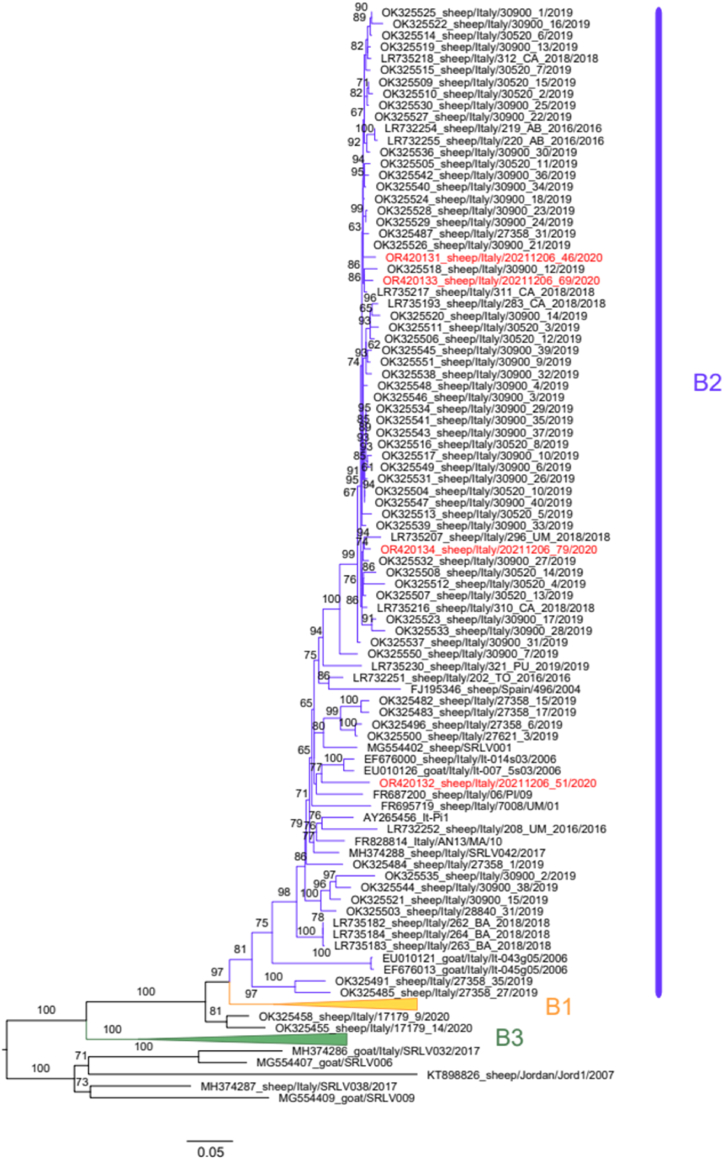
Maximum likelihood phylogenetic tree of genotype B samples. Samples of this study are colored in red. The corresponding subtype is highlighted in purple (B2). To enhance visualization, other subtypes were collapsed. Only bootstrap values above 60 are displayed.

A total of 76 animals were analyzed, of which 25 were PCR-positive (800 bp fragment). After gel extraction, Sanger sequencing was performed to obtain 20 clean and analyzable sequences (3 from Laticauda and 17 from Bagnolese). Eleven sequences were obtained and deposited in GenBank (accession numbers: OR420124 - OR420134). Two phylogenetic trees with all sequences identified in this investigation are shown in Fig. 2, Fig. 3, including genotypes A and B samples, respectively.

Five sequences, OR420126, OR420127, OR420130, OR420128, and OR420129, were categorized under the A24 subtype and formed a monophyletic subclade supported by robust bootstrap values (Fig. 2). The highest similarity to available A24 samples ranged from 88.3 % to 88.99 %.

Among the five new A24 samples described in this study, samples OR420126, OR420130, OR420128, and OR420129 formed a small subgroup and displayed high similarity between them, ranging from 98.41 to 99.48 %. Sample OR420127 displayed a lower similarity with the other newly described A24 (ranging from 92.21 % to 93.27 %).

Upon comparison within this subclade, sample OR420127 exhibited the lowest similarity (ranging from 92.21 % to 93.27 %) with other recently identified A24 samples, whereas samples OR420126, OR420130, OR420128, and OR420129 showed higher similarity levels, ranging from 98.41 % to 99.48 %.

Finally, samples OR420124 and OR420125 clustered together, with a similarity of 95.5 % between them. Compared to previously characterized sequences, sample OR420124 showed the highest similarity to LR735214 (85.96 %), belonging to the A24 subtype. In contrast, sample OR420125 exhibited the highest similarity to OK325492 (86.8 %), classified as A9. Owing to their low similarity to other SRLV samples and the inferred phylogenetic topology, it is likely that their subtype remains unassigned at the moment and could potentially be classified as a novel subtype in the future. A noteworthy feature was observed in the amino acid sequences of these two samples, as they contained a distinctive amino acid motif (AGRKE) within variable region 2, which was not found in any other previously described Italian sequence. This unique motif adds to the genetic makeup and warrants further investigation to determine its potential significance in the context of SRLV genotypes.

Phylogenetic analyses of OR420133, OR420131, OR420134, and OR420132 consistently indicated their affiliation with the B2 subtype (Fig. 3). Among these, sequences OR420133, OR420131, and OR420134 exhibited a notably high similarity, ranging from 97.7 % to 97.9 %, whereas sequence OR420132 displayed a comparatively lower similarity, ranging from 91.7 % to 92.5 %.

The four B2 sequences exhibited considerable diversity, as was evident from the tree topology, wherein they appeared to be interspersed with the other B2 samples. Comparative analysis of publicly available B2 sequences reveals that sequences OR420133, OR420131, and OR420134 showed the highest similarity to sequences OK325526, OK325507, and OK325526, respectively, with similarity scores ranging from 98.61 % to 99.07 %. This strong correlation suggested a close relationship with the reference samples.

In contrast, sequence OR420132 displayed notably lower similarity to the previously sequenced samples. The highest similarity, at 93.05 %, is observed with the sample OK325539, indicating a distinct genomic profile compared with the other B2 sequences.

## 4 Discussion

Italy presents a relevant number of sheep and goat breeds. Italy has the largest number of local goat breeds among the European countries. The Domestic Animal Diversity Information System (DAD-IS) of FAO currently reports 47 breeds. Approximately half of these are at risk, and 11 are classified as endangered [33]. The DAD-IS lists approximately 70 sheep breeds present in Italy, of which a certain number are reported to be at risk or endangered. There is considerable interest in activities aimed at conserving endangered breeds at the European, national, and regional levels. The European Union Reference Centre for Endangered Animal Breeds (EURC-EAB) has recently been created. The focus of the EURC-EAB is to provide scientific and technical contributions for the preservation of endangered breeds and their genetic diversity.

A number of initiatives (Oengene project https://www.oengene.at/, GEH https://www.g-e-h.de/, ELBARN http://www.elbarn.net/elbarn/, RARE https://www.associazionerare.it/, and SAVE Home (en) - Save Foundation (save-foundation.net)) and studies [1,34] have been dedicated to the conservation of endangered animal breeds in Europe, and FAO has issued a specific documentation (In situ conservation of livestock and poultry (fao.org)). Different aspects related to the conservation of these domestic breeds have been discussed; however, to the best of our knowledge, the role of infectious diseases as risk factors has generally been considered only for endangered wild species [[35], [36], [37], [38], [39], [40]], and very few reports have specifically addressed the presence and role of endemic infectious illnesses in endangered domestic small ruminant breeds [[41], [42], [43]]. Although infectious diseases are not generally considered a primary factor causing the endangerment or extinction of wild species, it has recently been highlighted that infectious diseases can cause significant population declines in wild animal species, increasing population fragmentation, and reducing genetic variability (gene flow) [36,40]. Therefore, it is reasonable to believe that infectious diseases could threaten endangered domestic breeds. As previously mentioned, SRLV is a retrovirus that causes persistent infections in goats and sheep. Therefore, a vaccine is unlikely to be developed in the near future. The prevalence of SRLV (Visna/Maedi) is increasing worldwide [44]. Finally, the emergence and pandemic spread of SRLV has been recently investigated, highlighting the role of commerce and breeding in the worldwide diffusion of the disease [45].

These observations prompted us to use a preliminary approach to investigate the presence of infection among endangered sheep and goat breeds in the Campania region using serological and molecular tests. A commercial ELISA (IDVet) and a recently developed in-house ELISA were used simultaneously for serological screening. The use of two or more tests in parallel has been recommended by several authors [3,46,47] due to the high genetic heterogeneity of these viruses, which can lead to an inaccurate diagnosis if a single test is used.

As reported above, in the present investigation, the in-house test gave 97 % of results in agreement with those found using the IDvet screening ELISA with a Cohen kappa = 0.84 (95 % CI = 0.76–0.93) [32]. Notably, the agreement between IDvet and another commercial test (IN3 Diagnostics screening ELISA) reported in a recent study was only fair (Cohen kappa = 0.21, 95 % CI = 0.16–0.26) [18] even if the IN3 Diagnostics test is credited with good sensitivity and specificity values [22]. According to a previous study, the Cohen's kappa found in the current investigation is considered very good for SRLV tests [48].

In the present study, discrepancies between the two tests were resolved by Western blot analysis. The in-house test correctly identified the infectious state of the herd in all the cases. Overall, we believe that the serological diagnostic approach used in the present study provides accurate and cost-effective results and can be applied in more extensive screening. A significant percentage of sheep was found positive, with an apparent prevalence of 12.5 % and 6.4 % in Bagnolese and Laticauda breeds, respectively. In Cilentana goats, the prevalence was residual (1.7 %). However, it is noteworthy that even relatively isolated herds were infected with the SRLV. Molecular analysis of 11 proviral partial sequences of 800 bp corresponding to the *gag-pol* encoding region allowed us to classify the circulating subtypes in the two herds as B2 and A24. The genotypes of two highly similar sequences remained unassigned. These could constitute new subtypes in the future.

## 5 Conclusion

In this study, we demonstrated the presence of SRLV in endangered small ruminant breeds with variable degrees of prevalence. In view of conservation efforts, it is important, as a second step, to extend screening to the entire population. In addition, we believe that according to the results obtained at the herd level, control/eradication of SRLV infection should be implemented in conjunction with each conservation program, considering the necessity of safeguarding the numeric consistency and genetic variability of each breed. Finally, the usefulness of screening endangered goat and sheep breeds for other “iceberg diseases,” such as paratuberculosis, border disease, and caseous lymphadenitis, should be evaluated.

## Data availability

GenBank (Accession numbers: OR420124–OR420134).

## Declaration

All procedures were approved by the Ethical Animal Care and Use Committee of the University of Naples Federico II (Prot. Nr. PG/2022/0146435 del 05/12/2022).

## CRediT authorship contribution statement

**Angela Ostuni:** Writing – review & editing, Visualization, Investigation. **Sara Albarella:** Writing – original draft, Visualization, Investigation. **Luca Tassoni:** Writing – original draft, Investigation. **Mariagiulia Pugliano:** Investigation. **Emanuele D'Anza:** Investigation. **Maria Antonietta Crudele:** Investigation, Formal analysis, Data curation. **Francesca Ciotola:** Writing – review & editing, Investigation. **Maria Serena Beato:** Writing – original draft, Investigation. **Valentina Iovane:** Investigation. **Stefano Cecchini Gualandi:** Investigation. **Raffaele Frontoso:** Investigation. **Jolanda De Vendel:** Investigation. **Vincenzo Peretti:** Investigation. **Alfonso Bavoso:** Writing – review & editing, Writing – original draft, Project administration, Methodology, Conceptualization.

## Declaration of competing interest

The authors declare that they have no known competing financial interests or personal relationships that could have appeared to influence the work reported in this paper.
