# Supplementary material for: Circulation of small ruminant lentivirus in endangered goat and sheep breeds of Southern Italy
Source: Heliyon. 2024 Jun 28;10(13):e33906. doi: 10.1016/j.heliyon.2024.e33906 (PMC11255564; doi:10.1016/j.heliyon.2024.e33906)
Supplement: Multimedia component 1 [file mmc1.pdf]

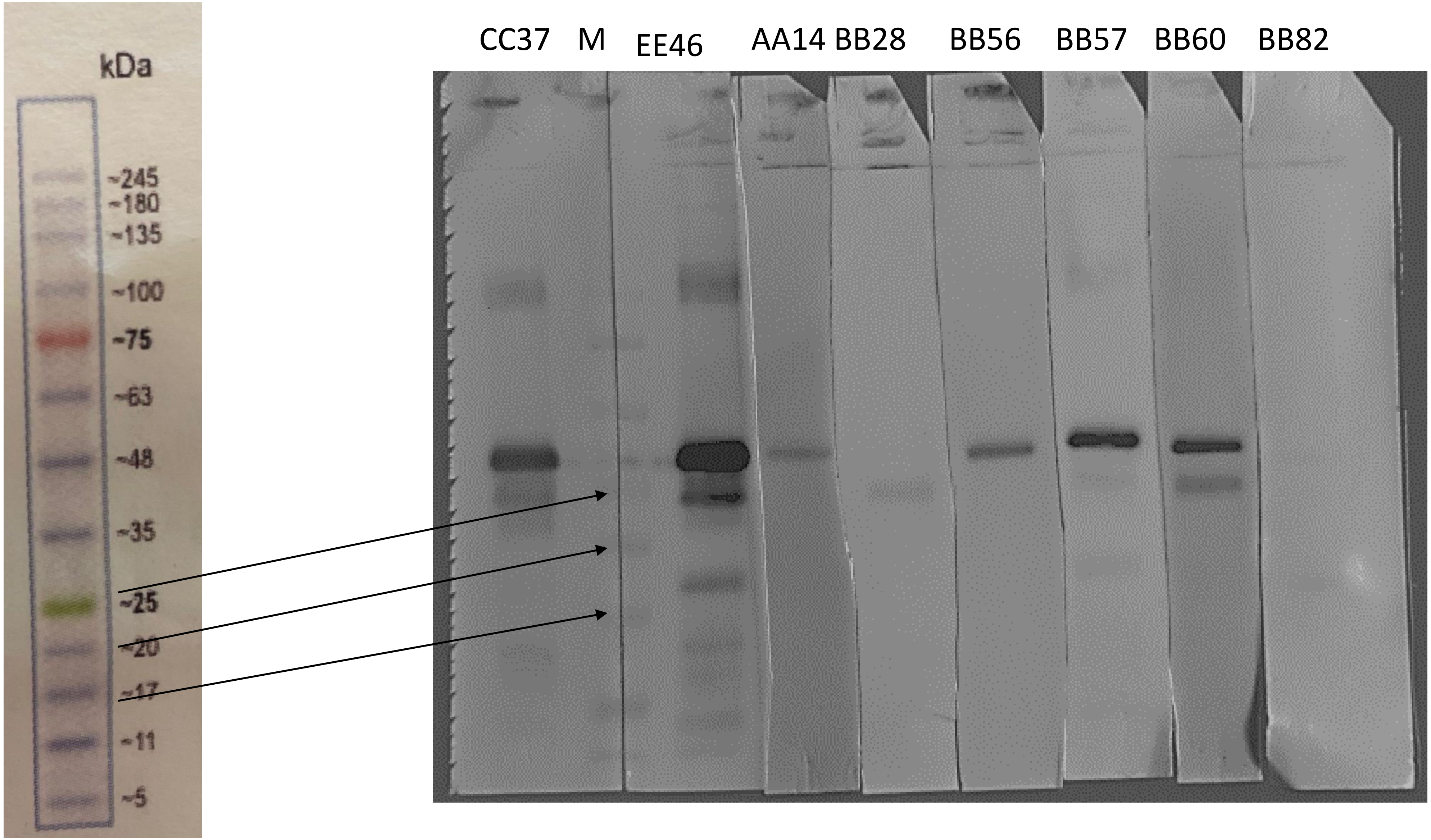

Original blot of Figure 1B. Arrows indicate molecular weight of Prestained Protein Sharp Mass VI Protein MW (5-245 Da) Marker (Euroclone). Each strip of the membrane (on which the three proteins P25_1, P25_frag2 and TM1_TM2 were immobilized) was incubated with a single serum corresponding to the goat indicated with the acronyms CC37, EE46, AA14, BB28, BB56, BB57, BB60, BB82. The image was captured by bringing the strips closer to each other.

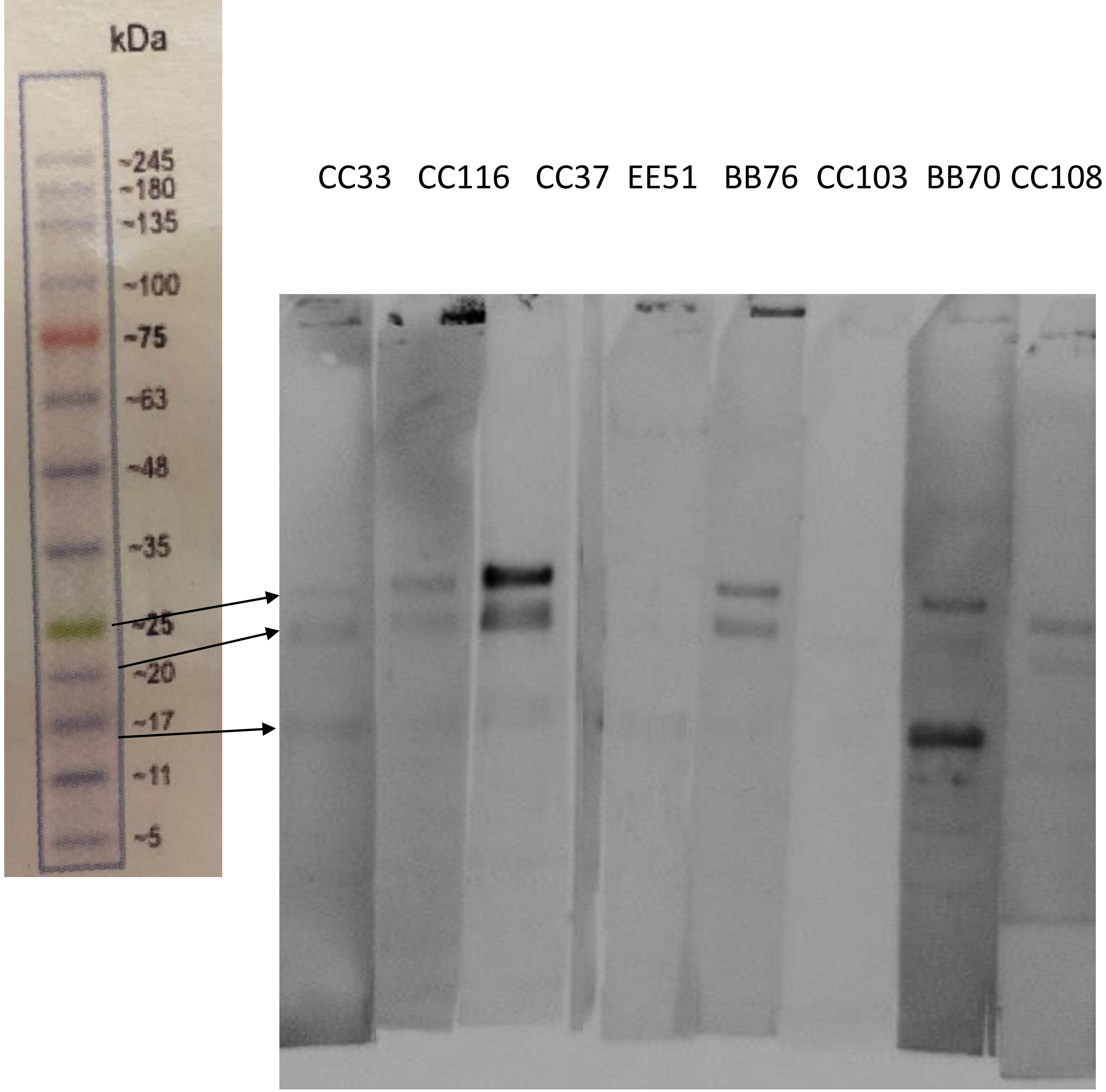

Original blot of Figure 1A. Arrows indicate molecular weight of Prestained Protein Sharp Mass VI Protein MW (5-245 Da) Marker (Euroclone). Each strip of the membrane (on which the three proteins P25_1, P25_frag2 and TM1_TM2 were immobilized) was incubated with a single serum corresponding to the goat indicated with the acronyms CC33, CC116, CC37, EE51, BB76, CC103, BB70, CC108. The image was captured by bringing the strips closer to each other
